# Supplementary material for: Climatic niche properties shape treefrog diversity
Source: PLoS One. 2026 May 6;21(5):e0348700. doi: 10.1371/journal.pone.0348700 (PMC13148696; doi:10.1371/journal.pone.0348700)
Supplement: S1 Table — Variable loadings for the three principal components. (DOCX) [file pone.0348700.s001.docx]

**S1 Table. Results of Principal Component Analysis**

**S1 Table:** Variable contribution for each principal component. The proportion of the variance for the first three components was: PC1= 0.615, PC2= 0.201 and PC3= 0.094.

| **Variable** | **PC1** | **PC2** | **PC3** |
| --- | --- | --- | --- |
| wc2.1_2.5m_bio_1 | 0.31318535 | -0.14317717 | -0.12207304 |
| wc2.1_2.5m_bio_10 | 0.27046359 | -0.1616663 | -0.30031539 |
| wc2.1_2.5m_bio_11 | 0.31757608 | -0.13053558 | -0.04429904 |
| wc2.1_2.5m_bio_12 | 0.27513548 | 0.27594564 | 0.10745292 |
| wc2.1_2.5m_bio_13 | 0.28263836 | 0.14146276 | 0.27458891 |
| wc2.1_2.5m_bio_14 | 0.14982724 | 0.45640367 | -0.25512363 |
| wc2.1_2.5m_bio_15 | 0.04086997 | -0.41133537 | 0.47377521 |
| wc2.1_2.5m_bio_16 | 0.28221887 | 0.15060304 | 0.26778819 |
| wc2.1_2.5m_bio_17 | 0.16247721 | 0.4548993 | -0.23900901 |
| wc2.1_2.5m_bio_2 | -0.01014146 | -0.38488571 | -0.44198951 |
| wc2.1_2.5m_bio_3 | 0.30443501 | -0.10017209 | 0.10426659 |
| wc2.1_2.5m_bio_4 | -0.30793021 | 0.10051125 | -0.08547359 |
| wc2.1_2.5m_bio_5 | 0.23880125 | -0.23145309 | -0.38875093 |
| wc2.1_2.5m_bio_6 | 0.31985608 | -0.10238257 | -0.02325672 |
| wc2.1_2.5m_bio_7 | -0.30858138 | 0.02691877 | -0.14971393 |
