# Supplementary material for: Climatic niche properties shape treefrog diversity
Source: PLoS One. 2026 May 6;21(5):e0348700. doi: 10.1371/journal.pone.0348700 (PMC13148696; doi:10.1371/journal.pone.0348700)
Supplement: S2 Table — Statistical fit and comparison of four evolutionary models of ancestral state reconstruction. (DOCX) [file pone.0348700.s002.docx]

**S2 Table. Ancestral reconstruction of niche centroids**

**S2 Table**: Evolutionary models used to reconstruct ancestral niche centroids. Brownian motion (BM), Ornstein-Ühlenbeck (OU), Early Burst (EB), and Rate Trend (RT). ** Selected model with lowest AIC.

| **Model** | **Pc1** | | **Pc2** | | **Pc3** | | **Temperature centroid** | | **Precipitation centroid** | |
| --- | --- | --- | --- | --- | --- | --- | --- | --- | --- | --- |
|  | **AIC** | **Weights** | **AIC** | **Weights** | **AIC** | **Weights** | **AIC** | **Weights** | **AIC** | **Weights** |
| **BM** | 1232.665 | 1.0 E-7 | 1535.112 | 3.2E-21 | 1082.204 | 3.4 E-18 | 2117.602 | 3.1 E-27 | 4334.515 | 5.1E-17 |
| **OU**** | 1200.457 | 0.996 | 1441.583 | 1 | 1001.761 | 1 | 1995.569 | 1.0 | 42297.508 | 1.0 |
| **EB** | 1236.436 | 2.5 E-8 | 1544.7 | 3.4E-23 | 1107.931 | 8.8 E-24 | 2557.026 | 1.2E-122 | 5572.398 | 2.4 E-292 |
| **RT** | 1212.034 | 0.003 | 1500.619 | 1.3E-13 | 1048.593 | 6.7 E-11 | 2073.744 | 1.0E-17 | 4287.074 | 3.1 E-13 |
