# Supplementary material for: Climatic niche properties shape treefrog diversity
Source: PLoS One. 2026 May 6;21(5):e0348700. doi: 10.1371/journal.pone.0348700 (PMC13148696; doi:10.1371/journal.pone.0348700)
Supplement: S1 Fig — Distribution and concentration of species with less than five occurrences. (DOCX) [file pone.0348700.s003.docx]

**S1 Figure. Location of non-modeled species**

The most contrasting differences in richness were found in the Atlantic Forest and Amazon regions, where most species could not be modeled due to insufficient presence points, but where IUCN polygons are available.


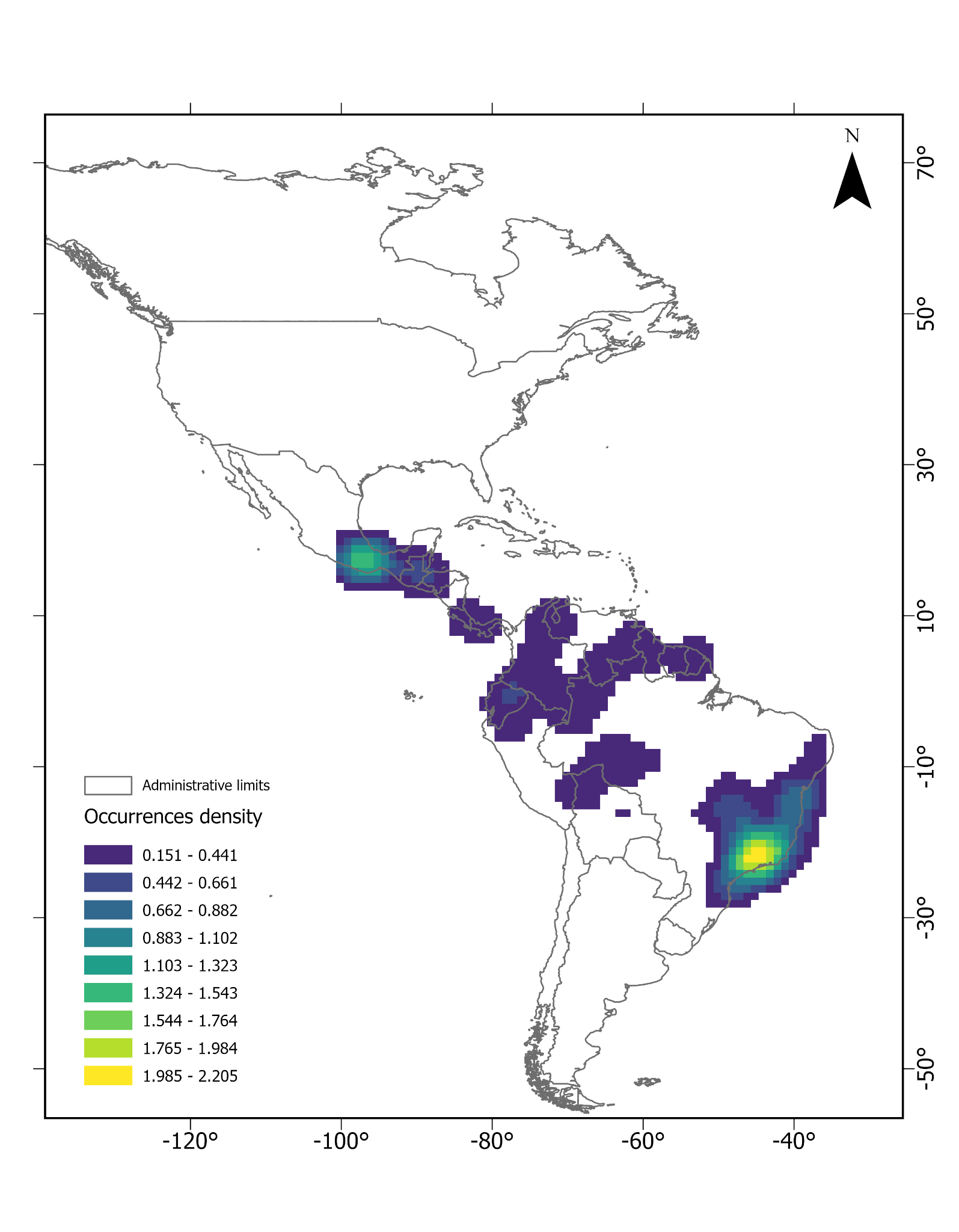


**S1 Figure.** Kernel density analysis of non-modeled species due to the low number of occurrences (n<5). Middle and high-density areas are shown in green and yellow colors. Administrative country limits are reprinted from GADM version 4.1 under a CC BY license, with permission from https://gadm.org/license.html, original copyright 2018.
