# Supplementary material for: Climatic niche properties shape treefrog diversity
Source: PLoS One. 2026 May 6;21(5):e0348700. doi: 10.1371/journal.pone.0348700 (PMC13148696; doi:10.1371/journal.pone.0348700)
Supplement: S2 Fig — Geographic prevalence of temperature and precipitation values in the study area. (DOCX) [file pone.0348700.s004.docx]

**S2 Figure. Distribution of temperature and precipitation values in the Americas**


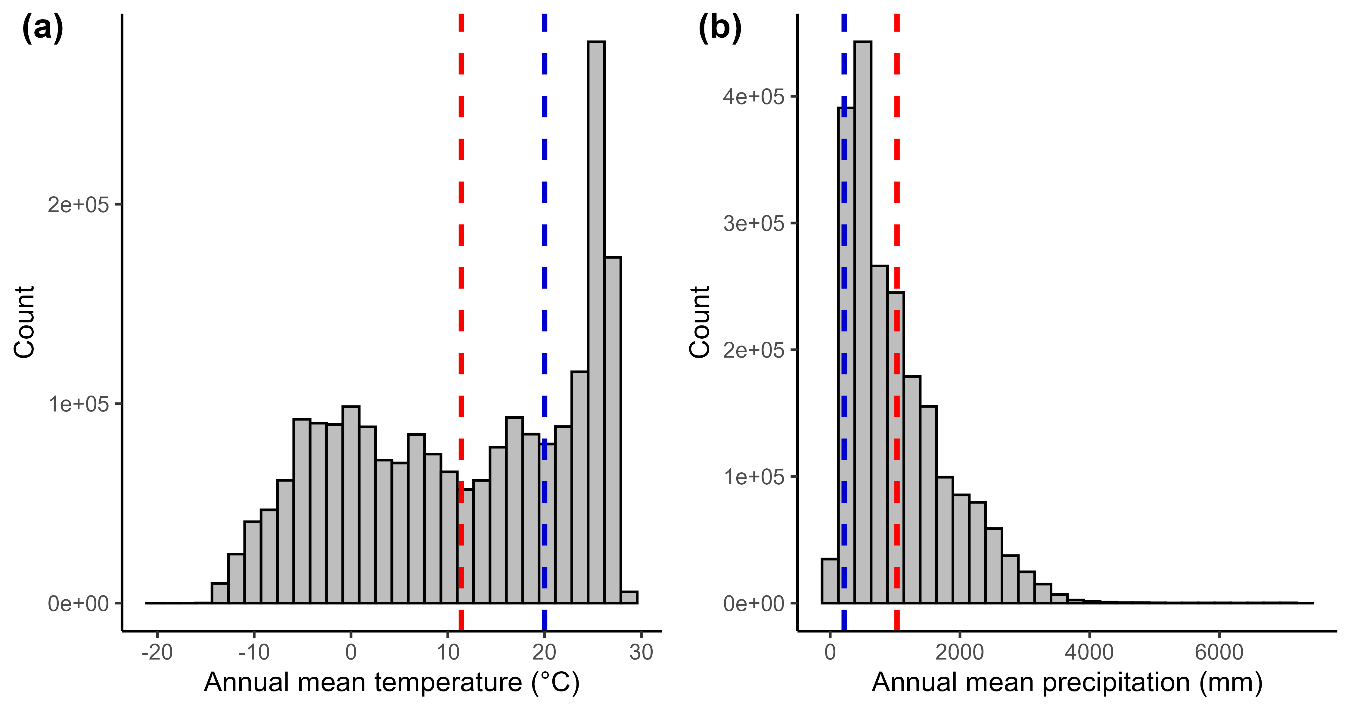


**S2 Figure.** Distribution of temperature (a) and precipitation (b) values. Red dashed lines represent the average values in the region of analysis, used to estimate marginality. Blue dashed lines represent the ancestral reconstruction of temperature and precipitation centroids, used to estimate position.
