# Supplementary material for: Climatic niche properties shape treefrog diversity
Source: PLoS One. 2026 May 6;21(5):e0348700. doi: 10.1371/journal.pone.0348700 (PMC13148696; doi:10.1371/journal.pone.0348700)
Supplement: S1 File — Ranking and distribution of species’ niche properties. (DOCX) [file pone.0348700.s005.docx]

**S1 File. Climatic niche properties of American treefrogs**

We found that species with larger niche breadth under the multivariate approach were not necessarily the ones with the largest temperature or precipitation breadth under the univariate approach. This situation applied to all niche properties (Fig. 3). We found that *Boana rubracyla* was the species with the most marginal niche but the least marginal precipitation. This same species is the most distant from its ancestral niche based on the multidimensional approach and for the precipitation ancestral centroid. Regarding the estimation of niche properties, our results showed little agreement between the species with the maximum and minimum rankings under the two approaches. For example, the species with the narrowest niche under the multivariate approach, or for temperature or precipitation under the unidimensional approach were *Boana caipao*, *Lysapus laveis* and *Pseudacris kalmi*, respectively, which have small distributions. On the other hand, the only agreement we obtained between approximations was for *Boana rubracyla*, which showed the largest distances to the ancestral centroid (largest position) of precipitation and ellipsoids. This could be explained by the fact that the species is distributed in the Pacific region of Colombia, which has the highest average annual precipitation of the continent.


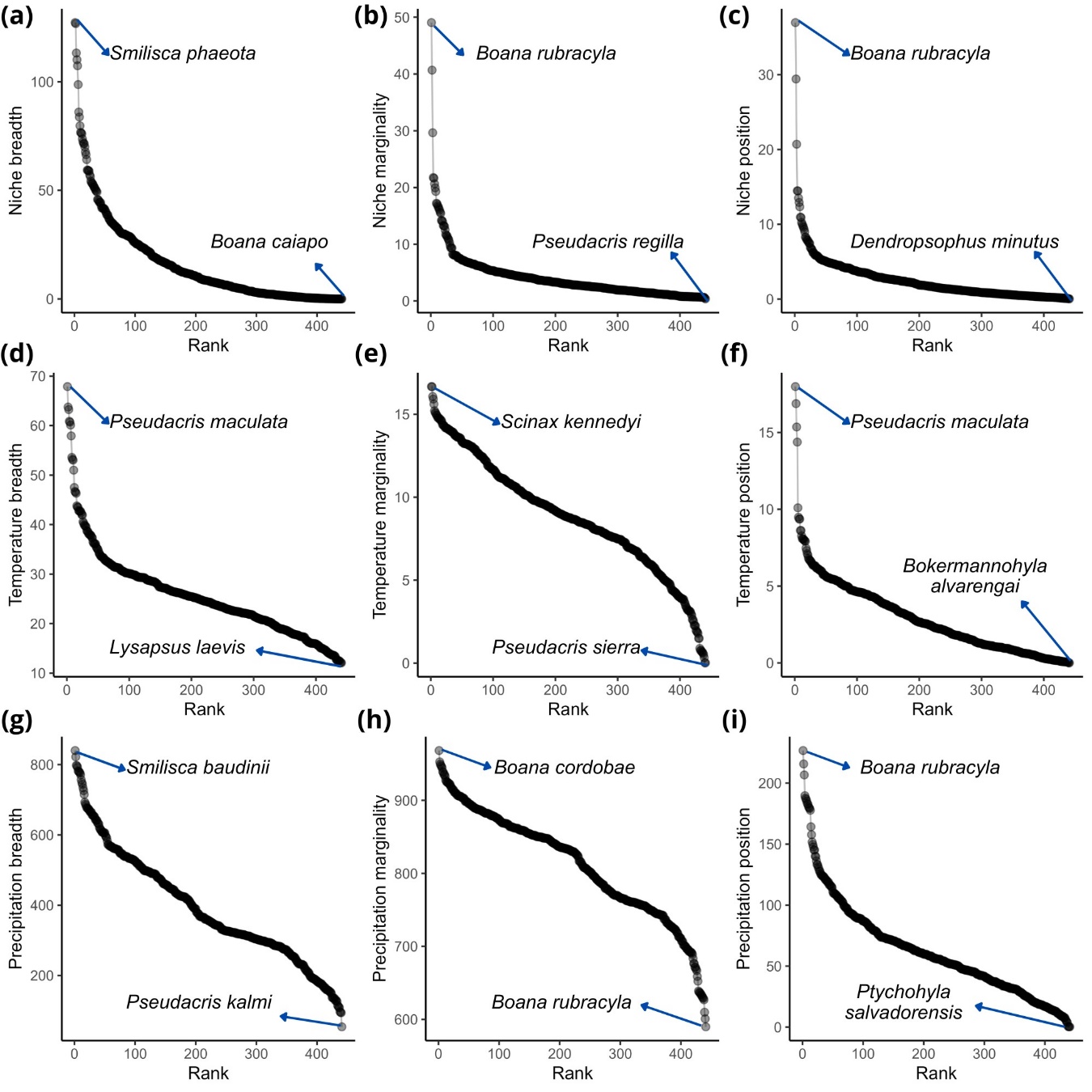


**S1 File Figure 1.** Ranking of species niche properties. a) Niche breadth; (b) Niche marginality; (c) Niche position; (d) Temperature breadth; (e) Temperature marginality; (f) Temperature position, (g) Precipitation breadth; (h) Precipitation marginality; (i) Precipitation position. Low ranks indicate the species with the highest value of each niche property, while major ranks correspond to species with the lowest values.

**
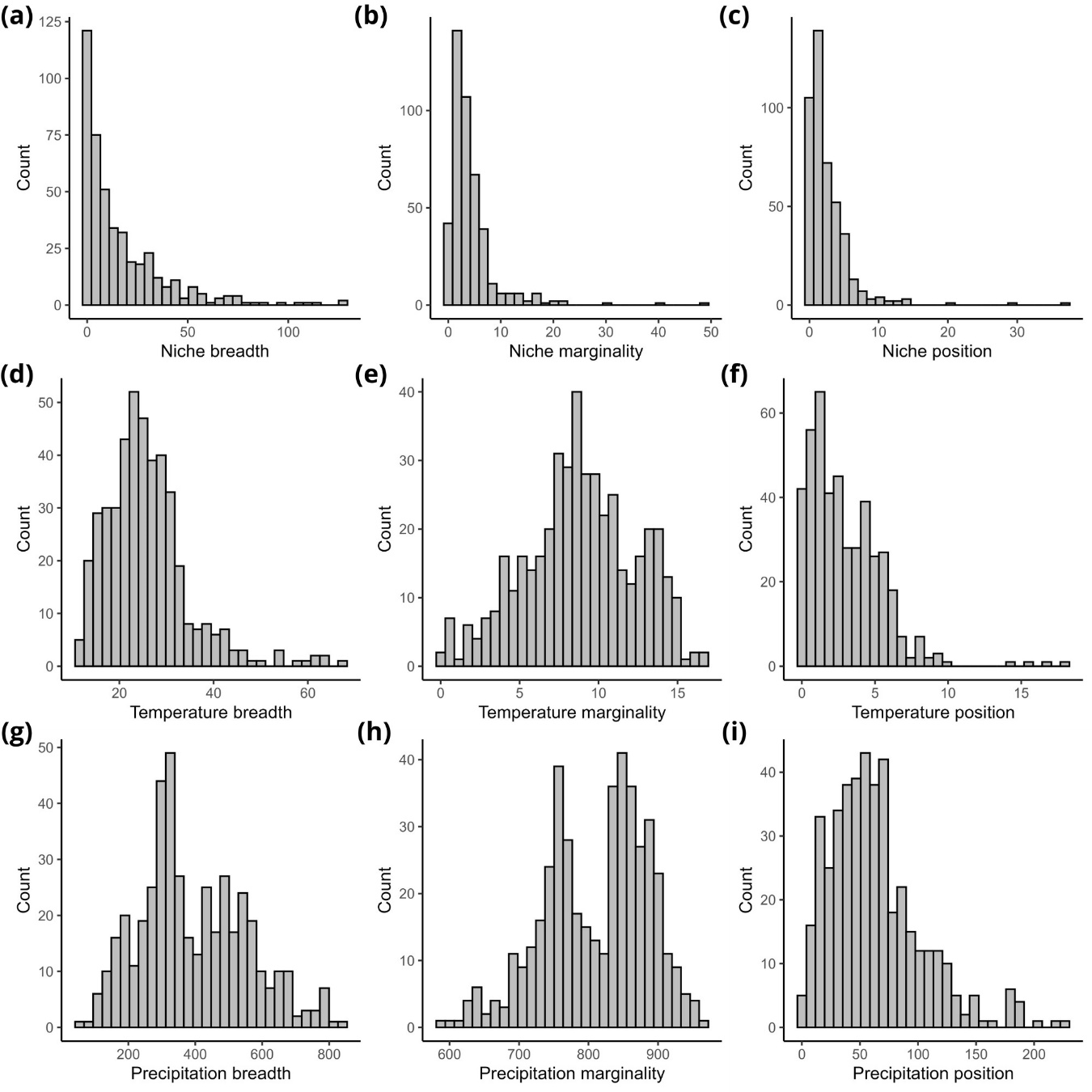
**

**S1 File Figure 2.** Histograms of species niche properties. (a) Niche breadth; (b) Niche marginality; (c) Niche position; (d) Temperature breadth; (e) Temperature marginality; (f) Temperature position, (g) Precipitation breadth; (h) Precipitation marginality; (i) Precipitation position.
