# Supplementary material for: Climatic niche properties shape treefrog diversity
Source: PLoS One. 2026 May 6;21(5):e0348700. doi: 10.1371/journal.pone.0348700 (PMC13148696; doi:10.1371/journal.pone.0348700)
Supplement: S2 File — Geographic areas with similar environments to the ancestral niche position and distribution of environments in the study area in relation to the ancestral niche position. (DOCX) [file pone.0348700.s006.docx]

**S2 File.** **Areas and climatic conditions close to the ancestral position using the multivariate approach**

We estimated the mahalanobis distances from the ancestral niche (pc1: 2.99, pc2: 0.85, pc3: 0.366) to the set of principal components at any site (see repository). Then, we calculated the 5th percentile of the distances and extracted the climatic values of the mean annual temperature (bio1) and mean annual precipitation (bio12). Later, we mapped the areas with climatic conditions close to the ancestral niche and performed histograms of bio1 and bio12. Most of the areas close to the ancestral niche centroid were found in the tropics (Fig 1) and corresponded to a mean temperature of ~25°C and mean precipitation of ~2000 mm (Fig 2).


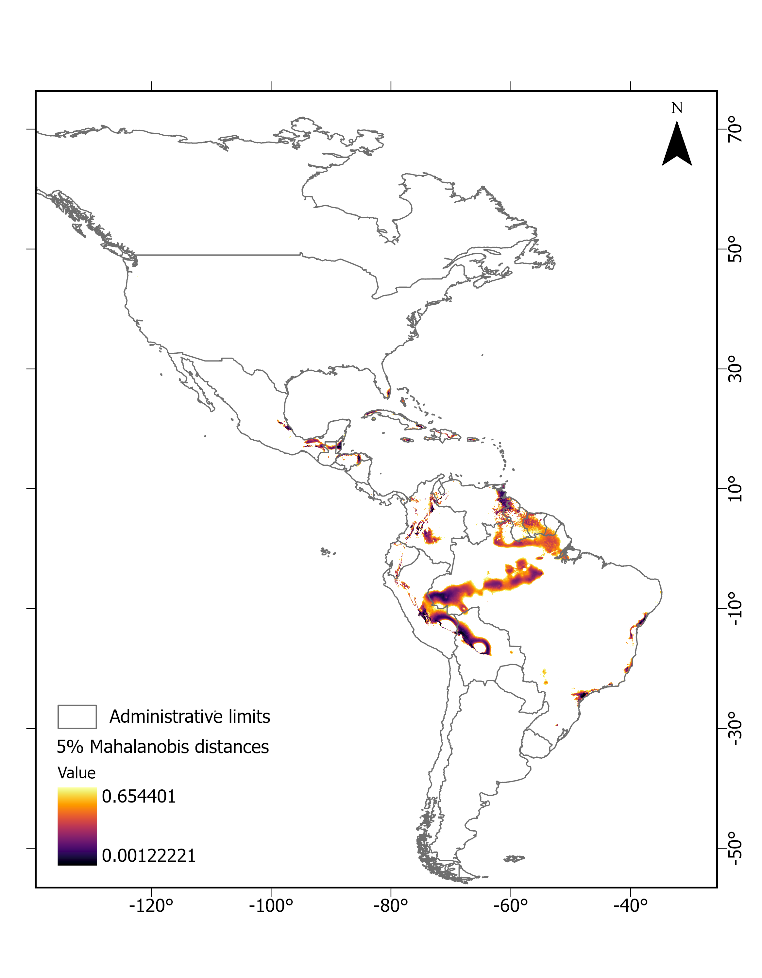


**S2 File Fig 1.** Geographical distribution of the 5th percentile of mahalanobis distances between ancestral centroid and environmental conditions using the principal component layers. Administrative country limits are reprinted from GADM version 4.1 under a CC BY license, with permission from https://gadm.org/license.html, original copyright 2018.


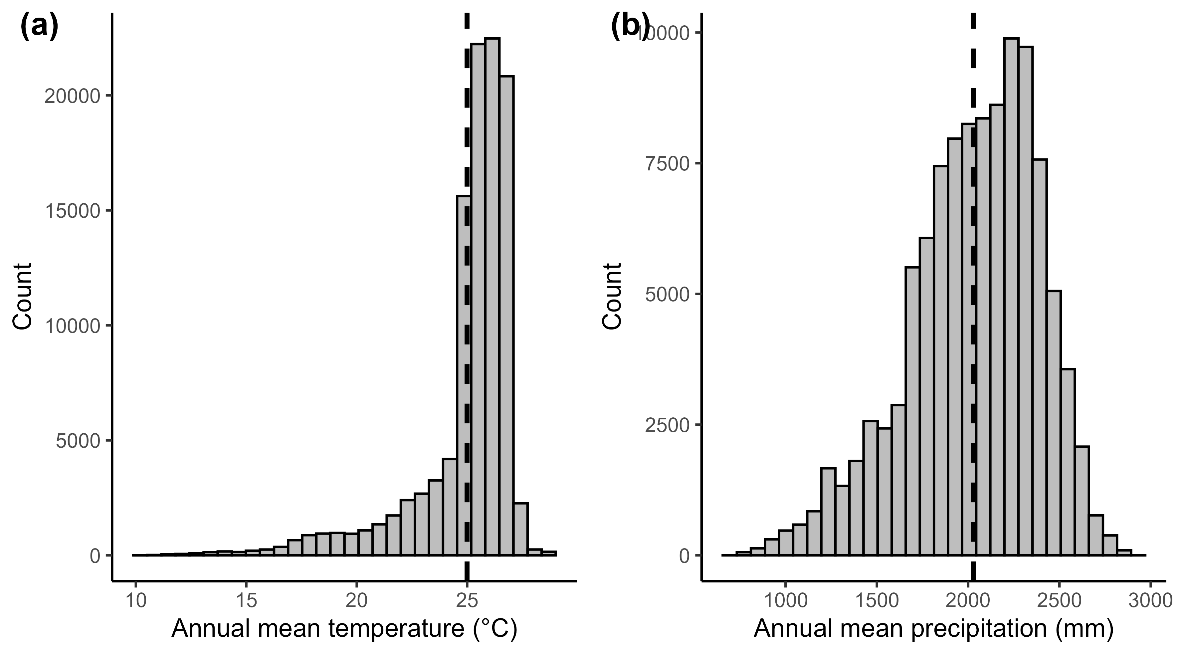


**S2 File Fig 2.** Distribution of mean annual temperature (a) and mean annual precipitation (b) values associated to the 5th percentile of mahalanobis distances between ancestral centroid and environmental conditions using the principal component layers. Black dashed lines represent the mean of the distribution values.
