# Supplementary material for: Climatic niche properties shape treefrog diversity
Source: PLoS One. 2026 May 6;21(5):e0348700. doi: 10.1371/journal.pone.0348700 (PMC13148696; doi:10.1371/journal.pone.0348700)
Supplement: S3 File — Statistical fit of various spatial autocorrelation models on climatic niche properties. (DOCX) [file pone.0348700.s007.docx]

**S3 File. Spatial autocorrelation and evaluation of SAR models**

**S3 File Table 1.** Evaluation of different SAR model configurations to explain species richness using climatic niche properties under the multidimensional approach. Schemes of the spatial weight matrix, W: row standardized, S: variance stabilizing; C: Global standardized. S^2^: Sigma square.

| Distance | Scheme | log likelihood | S^2^ | AIC | AIC OLS | I Morans |
| --- | --- | --- | --- | --- | --- | --- |
| Min* | S | -6393.31 | 3.48 | 12798.61 | 19763.97 | 0.97 |
| Max | S | -6474.19 | 4.30 | 12960.37 |  | 0.97 |
| Min | C | -6595.42 | 4.11 | 13202.83 |  | 0.98 |
| Min | W | -6634.46 | 4.12 | 13280.92 |  | 0.97 |
| Max | W | -6659.43 | 4.89 | 13330.86 |  | 0.96 |
| Max | C | -6721.45 | 5.22 | 13454.89 |  | 0.97 |

**
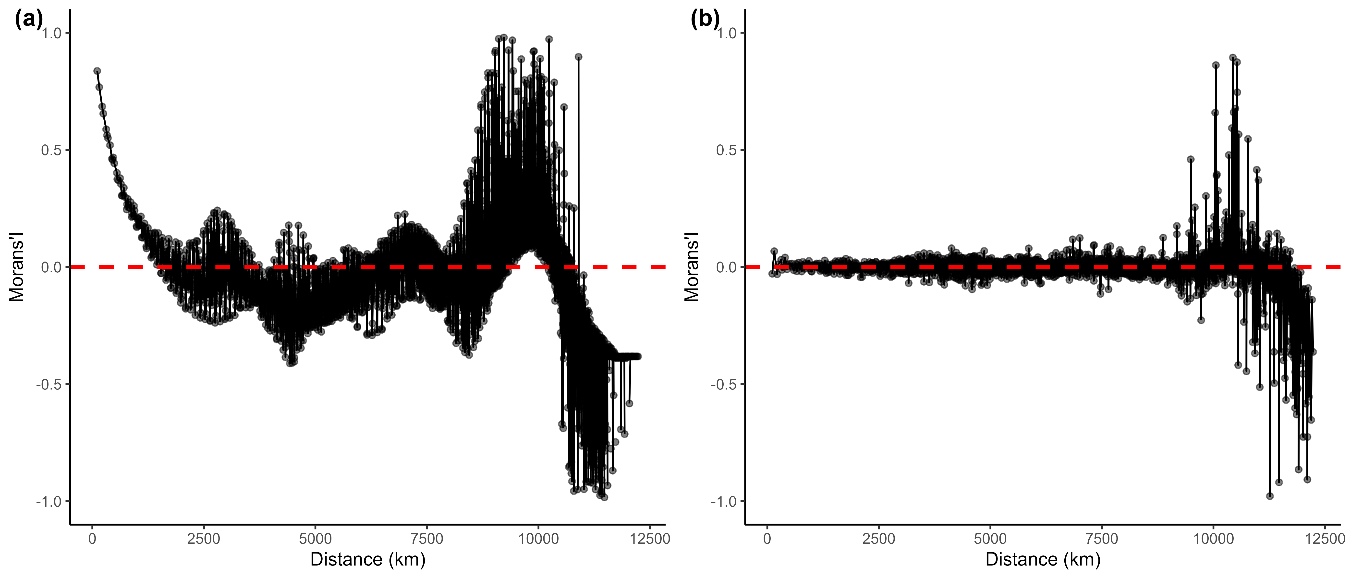
**

**S3 File Fig 1.** Spatial autocorrelation corrected by SAR models using ellipsoid properties. **(a)** OlS regression; **(b)** Minimum distance – variance standardized.

**S3 File Table 2.** Evaluation of different SAR model configurations to explain species richness using temperature properties. Schemes of the spatial weight matrix, W: row standardized, S: variance stabilizing; C: Global standardized. S^2^: Sigma square.

| Distance | Scheme | log likelihood | S^2^ | AIC | AIC OLS | I Morans |
| --- | --- | --- | --- | --- | --- | --- |
| Min* | S | -6484.43 | 3.70 | 12980.86 | 20237.46 | 0.97 |
| Max | S | -6596.04 | 4.69 | 13204.09 |  | 0.97 |
| Min | C | -6671.83 | 4.33 | 13355.67 |  | 0.98 |
| Min | W | -6776.53 | 4.54 | 13565.06 |  | 0.97 |
| Max | C | -6823.27 | 5.61 | 13658.54 |  | 0.97 |
| Max | W | -6827.11 | 5.47 | 13666.22 |  | 0.96 |


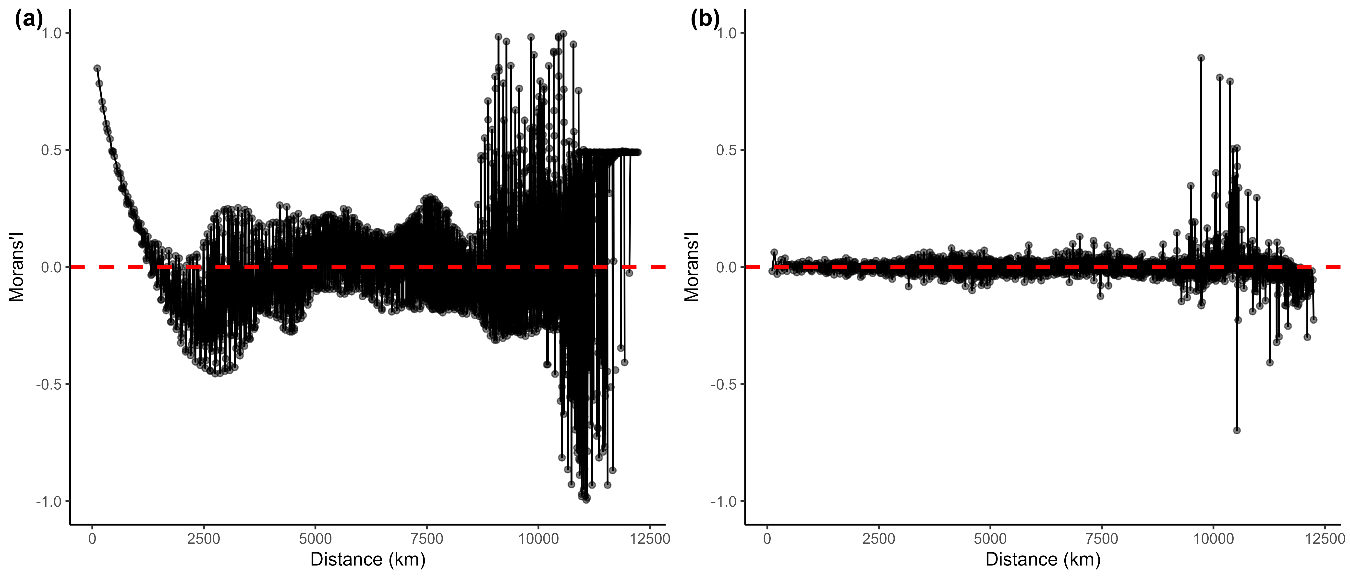


**S3 File Fig 2.** Spatial autocorrelation corrected by SAR models using temperature properties. (a) Ols regression; (b) Minimum distance – variance standardized.

**S3 File Table 3.** Evaluation of different SAR model configurations to explain species richness using precipitation breadth and position. Schemes of the spatial weight matrix, W: row standardized, S: variance stabilizing; C: Global standardized. S^2^: Sigma square.

| Distance | Scheme | log likelihood | S^2^ | AIC | AIC OLS | I Morans |
| --- | --- | --- | --- | --- | --- | --- |
| Min* | S | -6494.56 | 3.72 | 12999.12 | 21529.63 | 0.97 |
| Max | S | -6593.91 | 4.67 | 13197.82 |  | 0.96 |
| Min | C | -6723.87 | 4.48 | 13457.74 |  | 0.98 |
| Min | W | -6806.51 | 4.52 | 13623.02 |  | 0.96 |
| Max | W | -6832.84 | 5.42 | 13675.69 |  | 0.95 |
| Max | C | -6871.06 | 5.79 | 13752.12 |  | 0.97 |

**
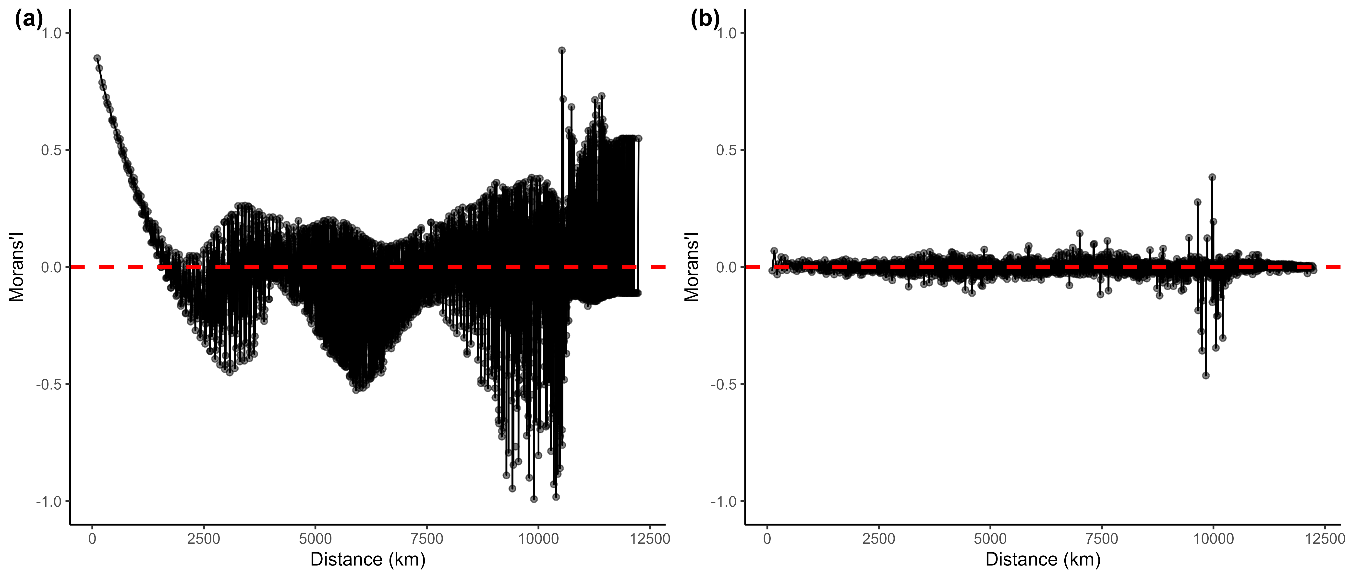
**

**S3 File Fig 3.** Spatial autocorrelation corrected by SAR models using precipitation properties. (a) Ols regression; (b) Minimum distance – variance standardized.

**S3 File Table 4.** Evaluation of different SAR model configurations to explain species richness using precipitation marginality and position. Schemes of the spatial weight matrix, W: row standardized, S: variance stabilizing; C: Global standardized. S^2^: Sigma square.

| Distance | Scheme | log likelihood | S^2^ | AIC | AIC OLS | I Morans |
| --- | --- | --- | --- | --- | --- | --- |
| Min* | S | -6497.27 | 3.72 | 13004.55 | 21439.84 | 0.97 |
| Max | S | -6598.50 | 4.69 | 13207.01 |  | 0.96 |
| Min | C | -6723.23 | 4.48 | 13456.46 |  | 0.98 |
| Min | W | -6811.83 | 4.54 | 13633.67 |  | 0.96 |
| Max | W | -6840.22 | 5.45 | 13690.45 |  | 0.95 |
| Max | C | -6871.33 | 5.79 | 13752.6 |  | 0.97 |


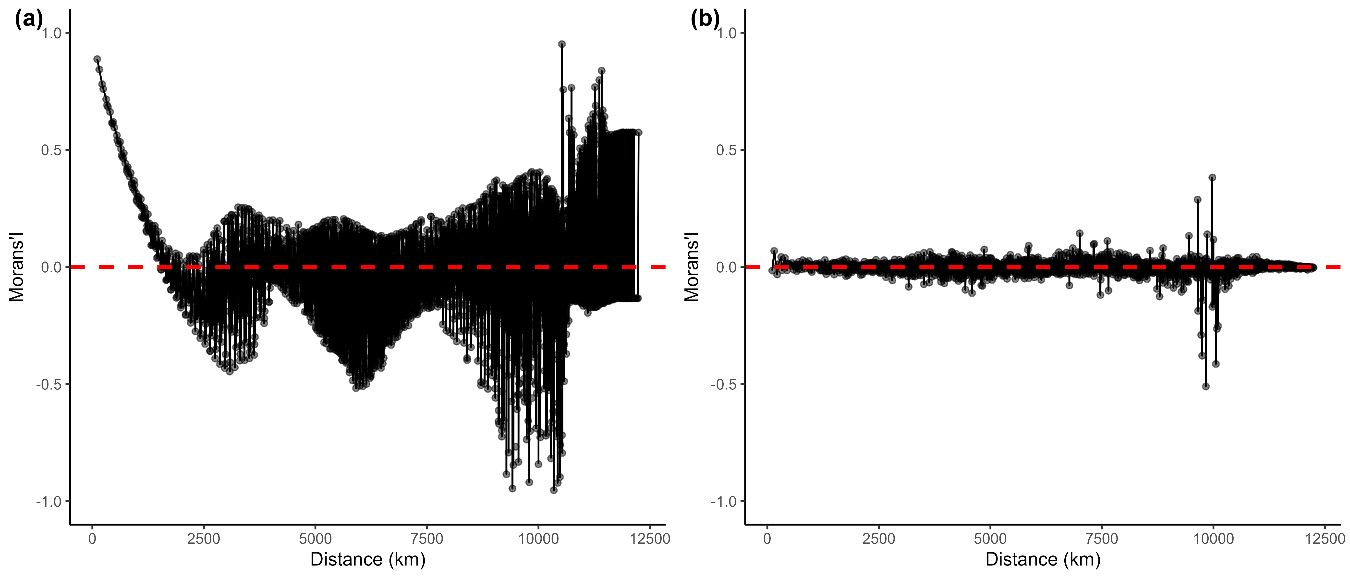


**S3 File Fig 4.** Spatial autocorrelation corrected by SAR models using precipitation properties. (a) Ols regression; (b) Minimum distance – variance standardized.
