# Supplementary material for: Climatic niche properties shape treefrog diversity
Source: PLoS One. 2026 May 6;21(5):e0348700. doi: 10.1371/journal.pone.0348700 (PMC13148696; doi:10.1371/journal.pone.0348700)
Supplement: S4 File — Distribution of estimated slopes between richness and climatic niche properties under two null models (geometric constraints and random distribution of mean niche properties). (DOCX) [file pone.0348700.s008.docx]

**S4 File. Geometric constraint and Null models results**

**
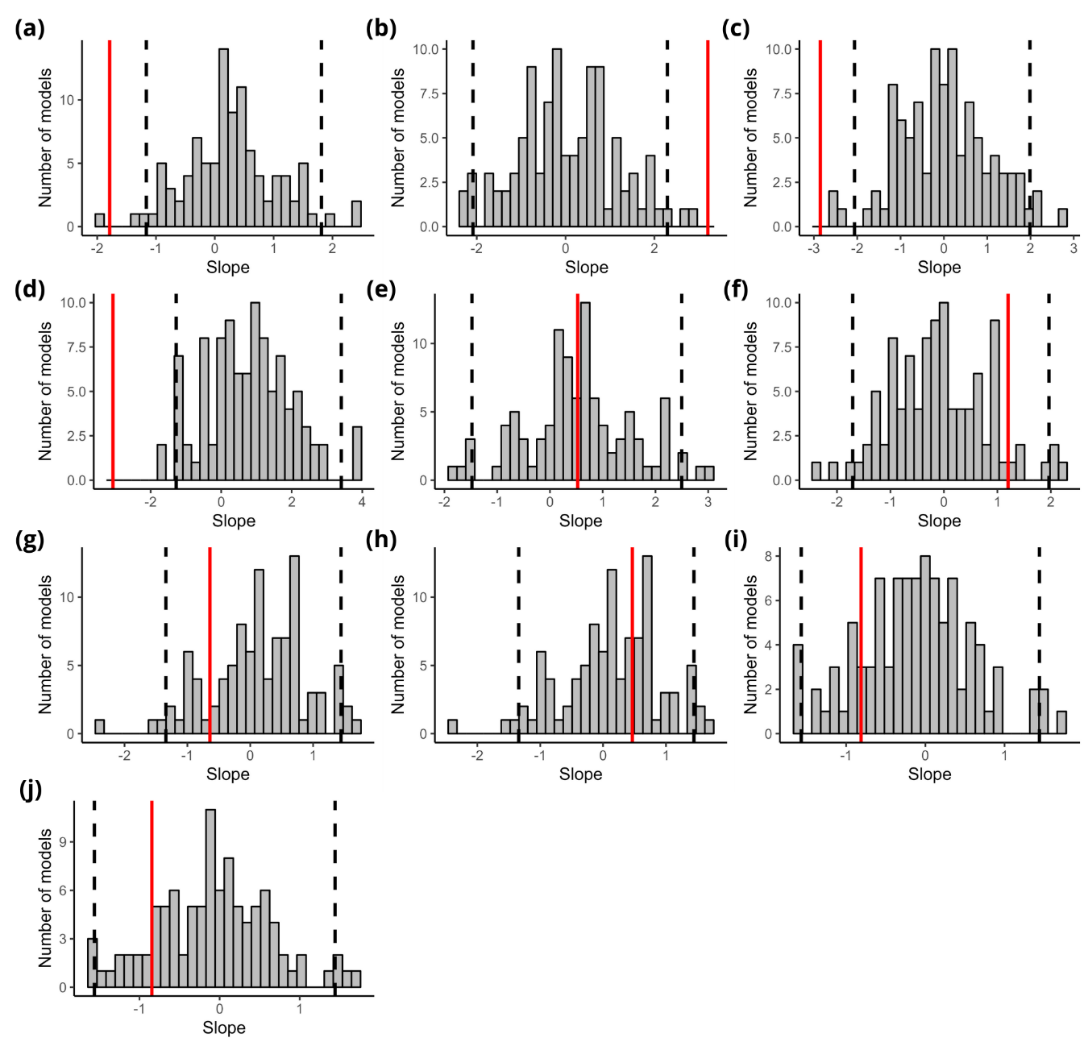
s**

**S4 File Fig 1.** Frequency distributions of geometric constraint model estimates across 100 simultaneous autoregressive models for species co-occurrences. Solid red line represents the observed value; black dashed lines represent 95% interval confidence of the null distribution. (a) Niche breadth; (b) Niche marginality; (c) Niche position; (d) Temperature breadth; (e) Temperature marginality; (f) Temperature position, (g) Precipitation breadth; (h) Precipitation marginality; (i) Precipitation position (PB+PP model); (j) Precipitation position (PM+PP model).

**
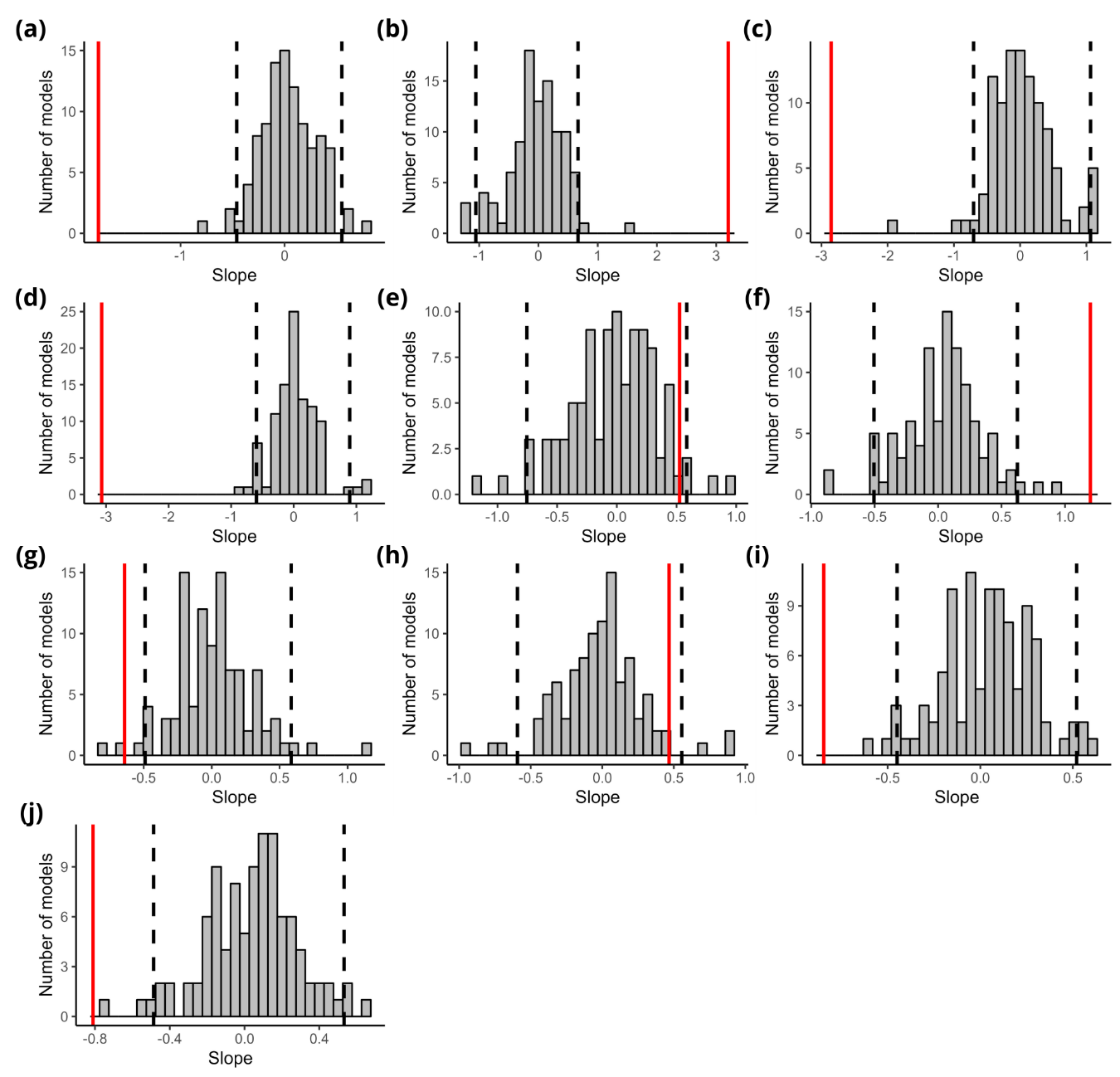
**

**S4 File Fig 2.** Frequency distributions of the null estimates across 100 simultaneous autoregressive models, using random distribution of niche properties. Solid red line represents the observed value; black dashed lines represent 95% interval confidence of the null distribution. (a) Niche breadth; (b) Niche marginality; (c) Niche position; (d) Temperature breadth; (e) Temperature marginality; (f) Temperature position, (g) Precipitation breadth; (h) Precipitation marginality; (i) Precipitation position (PB+PP model); (j) Precipitation position (PM+PP model).
