# Supplementary material for: Climatic niche properties shape treefrog diversity
Source: PLoS One. 2026 May 6;21(5):e0348700. doi: 10.1371/journal.pone.0348700 (PMC13148696; doi:10.1371/journal.pone.0348700)
Supplement: Attachment — Submitted filename: Response to Reviewers_R2.pdf [file pone.0348700.s011.pdf]

Climatic niche properties shape treefrog diversity

Response to reviewers' comments [6 march 2026]

Reviewer #1:

I appreciate the efforts made by the authors in addressing the reviewers' previous comments and suggestions. The manuscript has improved as a result. However, a few minor issues remain that should be addressed prior to final acceptance:

Line 106: The term "system" is not appropriate here, as anurans represent a biological group rather than a system. Please revise accordingly.
R: We changed "*system*" by "*group*".

Line 228: The acronym AIC is introduced here without prior definition. Please provide the full term at first mention.
R: Done.

Line 248: The acronym AIC is defined here; however, as noted above, this definition should be moved to its first occurrence in the text (line 228).
R: Done.

Line 256: The sentence begins with "These," yet only one null model has been described. Please rephrase for clarity and accuracy.
R: We changed "*These*" by "*This*".

Line 393: Please remove the parenthetical phrase "see below" and instead provide the relevant information directly at this point in the text.
R: We deleted the "*see below*" and mentioned a general problem, which is developed later in discussion. We now include the following sentences: "*The niche marginality property assumes that the most frequent environments in geography should be well represented by the mean. In our study area, the distribution of environments is bimodal (S8 Fig.), and thus the interpretation of the metric is not straightforward*".

Reviewer #2:

I thank the authors for their dedicated attention and for the careful and comprehensive implementation of the suggestions presented in the previous round of review; the changes made have fully addressed the issues raised, substantially enhancing the clarity, methodological rigor, and overall impact of the manuscript.

R: Thanks for your comments.
