# Supplementary material for: Climatic niche properties shape treefrog diversity
Source: PLoS One. 2026 May 6;21(5):e0348700. doi: 10.1371/journal.pone.0348700 (PMC13148696; doi:10.1371/journal.pone.0348700)
Supplement: Attachment — Submitted filename: Response to Reviewers_R1.docx [file pone.0348700.s010.docx]

**Climatic niche properties shape treefrog diversity**

**Response to reviewers’ comments**

**Reviewer #1:** 

This manuscript presents a compelling macroecological investigation into the latitudinal diversity gradient (LDG) of American treefrogs, using niche properties (breadth, marginality, and position) to explore hypotheses linking species-climate interactions to patterns of species richness. The study addresses an important topic and represents a valuable contribution to the field. However, to strengthen the manuscript, several areas require attention, particularly regarding methodological clarity, the consistency of the narrative, and the necessity of certain analyses. My specific comments below are offered to help the authors improve the rigor and impact of their work.

The manuscript would benefit from greater consistency in terminology. The core concepts are defined as niche breadth, marginality, and position. However, the text frequently introduces other theoretical names like "species-area" and "tropical niche conservatism" without clearly linking them to these core properties. This is confusing for the reader. I strongly recommend that the authors consistently use the terms "breadth," "marginality," and "position" throughout, and carefully justify how other hypotheses relate to these specific metrics.

R: We appreciate the reviewer’s suggestion. We have standardized the terminology in our manuscript and consistently use the terms breadth, marginality and position to avoid confusion. We clarified how these properties can be framed under other hypotheses or patterns.

In the abstract the phrase between lines 37-39 is confusing, is “precipitation” a single hypothesis?

R: We rephrased the sentence to make clear that we meant the niche position hypothesis, and that only variation in precipitation position under the univariate approach showed a pattern consistent with this hypothesis.

In the abstract the phrase between the lines 43-46 is also confusing. Please rephrase it.
R: We rephrased it as: *These results support the long-standing hypotheses of niche packing and niche conservatism, while suggesting that some niche dimensions are more constrained than others*.

Upon first mentioning the latitudinal diversity gradient (LDG) in line 50, the manuscript should briefly define this fundamental pattern. Please add a concise phrase to describe the LDG as the observed decrease in species richness from the tropics to the poles.

R: Done.

In lines 57-58, why are you using dashes?

R: We removed the dashes.

In line 77, the statement that "the most frequent climates in geography are expected to hold more species" requires justification. Providing a sentence or two of explanation is crucial for the reader to understand the foundation of your hypothesis.

R: The species area relationship is one of the oldest described patterns in ecology [22]. Various mechanisms have been associated with it, and it has been used as an alternative to explain patterns of species richness and climate [21]. The rationale behind is that if a certain climate is more prevalent in geography, it is expected to contain more species. We tried to explain this rationale as: *Average climatic conditions should represent those climates that are more prevalent in geography [21]. Following the species-area hypothesis [22], we expect that species richness should be higher in those climates [23]. Thus, the niche marginality hypothesis posits that species with low niche marginality (whose niche centroid is close to the average climatic conditions) should coincide with high richness areas with environments that are more prevalent, whereas species with high niche marginality should be concentrated in species-poor areas.*

In line 88, the rationale for using the "common ancestor of the clades" in the analysis requires explicit justification.

R: The tendency of species and clades to retain their niches and related ecological traits over time is called niche conservatism [26]. In the context of species richness distribution, this hypothesis predicts that species should concentrate in areas with climates similar to those of their ancestors. Phylogenetic niche position measures the environmental distance from each species niche to the niche of the most common ancestor. Under this hypothesis, we expect high richness areas to contain species with low phylogenetic niche positions.

The phrase on lines 105-106 appears to be disconnected from the main argument of the paragraph. Please either integrate it more smoothly by explaining its connection to the point being made, or consider moving it to a more appropriate location in the manuscript where it better fits the context.

R: We included two sentences at the beginning of the paragraph to smooth the transition between the ideas previously established.

Line 116, the word “and” is misplaced.

R: We rephrased the sentence to improve clarity and readability.

The Materials and Methods section requires significant clarification regarding the necessity of three specific analyses. Currently, their purpose is unclear, and as they are not discussed in the results, they detract from the manuscript's focus.

R: We appreciate the reviewer’s suggestion. We agree that presenting a large number of analyses detracts from the manuscript’s focus, and we have moved some of those results to the Supplementary Material. Nonetheless, we insist that all three mentioned analyses are justified and valuable in terms of providing reliability to our inferences. We explain the justification for each analysis below and have modified the text accordingly.

The MVE vs. IUCN richness comparison. The core methodology involves creating species ranges from MVE-derived niches. The rationale for comparing the resulting richness pattern to one derived from IUCN ranges is not explained. Since this comparison is not discussed, its inclusion seems unnecessary and should be justified or removed.

R: Thank you. We followed your recommendation and added the rationale behind this analysis as follows: “*Since we could not collect enough occurrence data to model all hylid species, we verified that the richness pattern obtained from our models (n=441 species) did not differ from the richness map obtained based on all expert range maps from IUCN (n=711 species) [50]. We correlated both richness estimates using a Spearman correlation test (data did not meet normality assumptions). Ellipsoid models exhibited consistent patterns (i.e., rho > 0.8) relative to those derived from IUCN range maps. Subsequent analyses were conducted based on the ellipsoid models.”*

We also added a discussion of the results from richness patterns obtained from niche models and IUCN polygons and their potential implications for mapping traits.

Multivariate vs. Univariate niche properties comparison: It is well-established that niche properties are multivariate. The justification for comparing these robust multivariate results to univariate approximations is lacking. This entire section is confusing and creates an inconsistent narrative. Without a clear purpose and discussion, its inclusion is difficult to support.

R: We estimated niche properties using both a univariate and a multivariate approach because each framework provides complementary insights. We made this explicit in the Methods Section, and we also address this in the Discussion Section. The univariate approach is the most widely used in macroecology, its inclusion offers a well-established basis for comparison with previous studies. In contrast, the multivariate approach is conceptually more appropriate for capturing the multidimensional nature of climatic niches, but it has not yet been thoroughly tested in macroecological or even macroevolutionary studies. By implementing both approaches, we intend to assess the robustness of our conclusions and to evaluate whether a multidimensional characterization of the niche yields additional insights into the drivers of large-scale richness patterns. We mentioned this in the last paragraph of our introduction

Null model analysis. The aim of the null model comparison is not clear. Furthermore, I must point out a conceptual issue: the "spreading-dye" model (or Geometric Constraint Model - GCM) is not a true null model but a mechanistic hypothesis that itself explains richness patterns through geometric constraints. The use and interpretation of this analysis require substantial clarification. I strongly advise the authors to either (1) provide a compelling a priori justification for each of these analyses and integrate a thorough discussion of their results into the manuscript, or (2) remove them entirely. I am inclined to recommend removal, as this would significantly improve the manuscript's clarity, focus, and logical flow.

R: We agree with the reviewer’s comment. In the new version of our manuscript, we explicitly distinguish between the Geometric Constraint Model, used here as a mechanistic reference hypothesis, and a randomization-based null model that tests for non-random spatial associations between niche properties and richness by shuffling species identities while holding richness constant. Furthermore, we distinguished both results and discuss them in Discussion section

Line 135, this “five-kilometers radius” has a justification?
R: We agree that the rationale behind the 5-km thinning needs further clarification. We applied this threshold because occurrences located within the same pixel (5 km resolution) share identical environmental information. In our case, the 5-km radius corresponds directly to the spatial resolution of the environmental grid and prevents the inclusion of multiple records that do not contribute additional environmental information but would increase spatial autocorrelation. This process also contributes to reducing both, spatial biases and model overfitting; hence improving model performance (Boria et al. 2014; https://doi.org/10.1016/j.ecolmodel.2013.12.012).

We agree that the selection of the threshold value may be subjective. In an ideal scenario, this value would correspond to home range size or maximum dispersal capacity; unfortunately, this information is scarce, especially for amphibians. Further, increasing the threshold value would reduce the number of species that meet the minimum number of occurrences required for niche modeling.

We added a sentence in the Methods Section specifying: “*We chose this threshold to meet the resolution of our environmental data; nonetheless, we believe this distance represents enough spatial separation to warrant occurrence records independent*”.

Line 173, “teste” is miswritten.
R: Corrected.

Lines 188-189, what are the environmental variables?
R: We added a sentence in the Methods Section giving a general explanation of the climatic variables provided in Worldclim: “*These variables represent general tendencies and annual temporal variation in temperature and precipitation regimes established over a ~30-year interval*”.

In lines 188-189 we made reference to the general method for estimating marginality. Then we explained the multivariate and univariate approaches. Variables for this last one are specified in the previous sentence of the paragraph. We clarified this as follows: *…Then, to estimate marginality in both individual dimensions, we calculated the Euclidean distance between each species centroid and the mean value of mean annual temperature (bio1=11.4°C) and annual precipitation in the study area (bio12=1029.7 mm).*

Line 191, “the environmental space” is based in what?
R: We clarified the phrase by specifying that the environmental space was based on the first three principal components out of all climate variables included.

Lines 193-199, this reading is very confusing, please rephrase.
R: We rephrased these lines to improve the clarity of the methods section. In this new version, we first explained the multivariate approaches for each niche property and then the univariate approach.

Line 201, “both approaches”? What approaches?
R: We clarified this in the first sentence of the “Treefrog climatic niche properties” Section: “*We calculated each niche property separately using the unidimensional and multidimensional approaches*”. We also justified why both approaches are informative.

Line 206-208, I do not understand what you want to say here. Please clarify.
R: We clarified this phrase as follows: “*We reconstructed the ancestral states of the niche centroids based on this phylogenetic hypothesis and the niche centroids estimated under the univariate and multivariate approaches for the current species*”.

Line 222, the GIS software, what GIS software?
R: We added the software used: ArcGIS Pro v. 3.5

Line 243-247, again, I did not understand what do you want to say here, please rephrase.
R: We rephased as follows: *The second null model evaluated whether the estimated relationships between predictors and observed richness differ from those obtained under a random distribution of mean niche properties in geography*

Line 264, Atlantic Forest and Amazon are biomes, Guiana is a country. Please rephrase.
R: We rephrased as follows: *Both datasets produced a congruent spatial pattern of richness, showing high diversity in the Neotropical region and lower diversity in temperate zones, with the Atlantic Forest, Amazon, and Guiana Shield standing out as richness hotspots*

Line 281, “extreme values” what do you want to mean with “extreme”?
R: We clarified this in the text: “*The following genera had at least one niche properties with* *minimum or maximum values out of all species considered: Boana, Dendropsophus, Smilisca, Scinax, Ptychohyla and Pseudacris. However, there was no consistency in the identity of species with the minimum or maximum value between approaches (unidimensional versus multidimensional) or niche dimensions (temperature versus precipitation; S4)*.”

Line 283, again, “extreme niche properties”, what do you want to mean with “extreme”?
R: Please see the previous comment.

Line 356-358, you are repeating results in the discussion.
R: We appreciate the reviewer’s comment. In our first paragraph in the discussion section, we aimed to briefly highlight our most relevant results which are deeply discussed in greater detail later. We consider that this is a useful way to contextualize the whole Discussion section.

Line 364, please delete the parentheses.
R: Done.

Line 372, the parentheses are repeating the results, please delete it.

R: Done.

Lines 378, 430, and 433, the citations are miswritten, the citations should be presented in numbers.
R: Corrected.

Lines 391-393, they are “distant” or “close” in relation of what?

R: We clarified the phrase as follows: *In our study area, the average temperature (11.4 °C) differs markedly from the most frequent temperature value (~26°C), whereas the average precipitation (1029.7 mm) is comparatively close to the most prevalent precipitation value (~850 mm).*

**Reviewer #2:**

The manuscript highlights the relevance of considering multiple dimensions of the climatic niche to explain macroecological patterns, offering important insights into the evolution and distribution of treefrog diversity. Thus, this article presents a significant contribution to ecology and biogeography by interestingly investigating how climatic niche properties—breadth, marginality, and phylogenetic position—influence treefrog diversity in the Americas.

**Reviewer #3:**

General comments

The manuscript presents important results, particularly in the comparison between unidimensional and multidimensional approaches, which provides a broader view of macroecological patterns. I recommend accepting the manuscript in PLOS ONE, after improving the writing to enhance clarity and objectivity in the presentation of results. My main criticisms are: (1) although the analyses show scientific rigor, in several sections the discussion does not seem fully aligned with the results obtained; (2) there is a lack of standardization in the use of terms (e.g., “marginality hypothesis” and “species-area hypothesis”), as well as in the order in which results are presented. Understanding improves when this order is consistent across sections; and (3) the text would be clearer if, instead of citing the hypotheses (e.g., “we found a negative relationship between niche breadth and species richness”), the authors cited the predictions (e.g., “species richness was higher where species exhibited narrower niches”). The hypotheses must be clearly described in the Materials and Methods, but the discussion becomes more objective when focused on the observed patterns. Below I provide suggestions for textual adjustments and more specific questions that need to be addressed.

Abstract
Lines 30–31: “the species-area hypothesis predicts higher richness in areas with more frequent climates.” I suggest standardizing the wording to facilitate understanding of each hypothesis. For example: “the marginality hypothesis predicts higher richness in areas with more common climatic conditions.” Additionally, the meaning of “more frequent climates” and how this relates to marginality is unclear. Are more frequent climates located near the center of the species’ climatic distribution? Clarify and standardize terminology.

R: Thanks for pointing this out. We have reviewed our wording and improved it by standardizing the terminology to “niche breadth hypothesis”, “niche marginality hypothesis” and “niche position hypothesis”. The relationship between marginality and climate frequency in geography is based on the species-area hypothesis. If certain climates are more geographically extensive, we expect those climates to host more species. Average climate conditions should be determined by the most frequent climates in geography, and thus, we expect species with low marginalities to be concentrated in areas with high richness. We explained this in the introduction section.

Lines 31–32: “the species-area hypothesis predicts higher richness in areas with more frequent climates.” Likewise, maintain the term “position” when explaining the hypothesis. For example: “the position hypothesis predicts higher richness in areas where species niches are closer to those of their ancestors.” Remember this is the abstract, and detailed explanation will appear in the Introduction.

R: Thank you for the suggestion, we maintained the niche position hypothesis in the abstract as you suggested.

Lines 32–33: “We estimate niche properties of all American treefrog species using a univariate...” Not all American species were analyzed, since some lacked sufficient occurrence records to build niche models.

R: We rephrased and specified the number of modeled species (441).

Lines 40–41: “We found no support for the species-area hypothesis under both approaches.” I suggest replacing with: “marginality hypothesis.”

R: Done.

Introduction

Lines 75–83: “Niche marginality... Hirzel et al. [20].”
This paragraph is unclear regarding the relationships among marginality, niche centroids, and regional climatic means. Under what conditions is the distance between a species’ niche centroid and the regional climatic mean smaller? Do species with low marginality exhibit high or low centroid-to-mean distance? Marginality relative to what exactly? Do species with high marginality have centroids closer to extreme deviations from the regional mean? I suggest reorganizing as follows: define what marginality measures, explain what constitutes high and low marginality, and then describe its expected relationship with richness.

R: We modified the marginality paragraph in accordance with the reviewer’s suggestions. We explain the rationale behind the hypothesis, its relationship to the species-area hypothesis, and why we expect that regions with high richness should contain species with low niche marginality values.

Lines 82–83: “but the original concept corresponds to marginality proposed by Hirzel et al.” What is the original concept proposed by Hirzel et al.? All essential information should be included in the text instead of requiring the reader to pause and search externally.

R: The original concept of marginality proposed by Hirzel et al. is now mentioned in the first line of this paragraph. We deleted the last sentence of the section to avoid confusion.

Lines 76–77: “In addition, the most frequent climates in geography are expected to hold more species.” The meaning of “more frequent climates” remains unclear, as does why they are expected to harbor more species.

R: We apologize for the confusion. By “more frequent climates”, we meant those climates that are more prevalent in geography. The relationship between climate frequency in geography and niche marginality is based on the species-area hypothesis.

Lines 117–118: “and a multivariate climatic approach: the niche-breadth, species-area, and tropical niche conservatism hypotheses.”
Again, I suggest standardizing the terminology. Choose whether to refer to the hypotheses as niche breadth, marginality, and position, or as niche breadth, species geographic range (species-area), and niche conservatism. In some parts, they appear to represent different hypotheses.
R: We standardized the terminology using the niche properties.

Table 1: The definition of marginality and how its numerical values indicate low marginality are still unclear.
R: =We clarified in the text (Lines 72–79) and in Table 1 the expectation under the niche marginality hypothesis: areas with high species richness should contain species with low marginality (niche centroid close to average climate conditions).

Materials and Methods

Lines 134–136: “We applied a spatial threshold of a five-kilometer radius between records to reduce spatial autocorrelation and obtain a single occurrence per pixel.”
It is not clear how a 5-km radius ensures one occurrence per pixel. What is the pixel size? If two pixels fall within the 5-km radius, is only one selected? Explain the steps of this criterion and the pixel grid definition.

R: We agree that the rationale behind the 5-km thinning needs further clarification. We applied this threshold because occurrences located within the same pixel (5 km resolution) share identical environmental information. In our case, the 5-km radius corresponds directly to the spatial resolution of the environmental grid and prevents the inclusion of multiple records that do not contribute additional environmental information but would increase spatial autocorrelation. This process also contributes to reducing both spatial biases and model overfitting; hence, improving model performance (Boria et al. 2014; https://doi.org/10.1016/j.ecolmodel.2013.12.012).

We agree that selecting the threshold value may be subjective. In an ideal scenario, this value would correspond to home-range size or maximum dispersal capacity; unfortunately, this information is scarce, especially for amphibians. Further, increasing the threshold value could reduce the number of species that present the minimum number of occurrence requirements for niche modeling available, reducing the number of species included in our analysis.

Lines 136–141: “Once we obtained... one occurrence record”.
Some sections are repetitive. I suggest the following revision:
“Once we obtained a manually curated database of occurrence records, we used it to delimit an area within the Americas where at least one treefrog species could occur. Because treefrogs are absent from extreme latitudes (e.g., northern Canada; southern Argentina and Chile), these regions were excluded. This area was delimited using the Tropical Nature Conservancy terrestrial ecoregions [39]”.
R: Done.

Lines 142–149: “We obtained the climatic variables... the same environmental space”.
This section belongs in the next subsection (Ecological niche modeling and species richness).
R: Since climatic variables are needed to generate the niche models and to estimate the niche properties, we opted to present them in the section that appears first in the text, which we titled “Species occurrences and environmental data”.

Lines 159–161: “Then, to obtain a species distribution... in all areas outside”.
Suggested revision:
“A species was considered present in all geographic areas whose climates fell within the ellipsoid, and absent in areas entirely outside the ellipsoid.”
R: Done.

Line 175: “we used a Spearman correlation test”.
Explain that the data did not meet normality assumptions, and therefore the Spearman correlation test was used.

R: Done.

Lines 175–177: “If the patterns exhibited consistency (i.e. rho > 0.8), subsequent analyses were conducted based on results from ellipsoid models”.
This sentence can be more direct and consistent with past-tense writing:
“Subsequent analyses were conducted based on the ellipsoid models that exhibited consistent patterns (i.e., rho > 0.8) relative to those derived from IUCN range maps.”

R: Done.

Lines 180–187: “We calculated each niche... wettest month (bio14)”.
I suggest standardizing the order of presentation, beginning with the unidimensional approach (first mentioned in Materials and Methods), followed by the multidimensional one. Apply this standardization across all sections.
R: We appreciate the reviewer’s suggestion. However, the structure of the manuscript was intentionally designed to present the multidimensional approach before the unidimensional one, as the former constitutes the central focus of our study, and conceptually, the niche is a multidimensional concept. In theory, the order in which we present the results should not affect our final interpretation. Nevertheless, we carefully reviewed the manuscript to ensure that the sequence of approaches is consistently presented throughout all sections.

Results
Lines 252–255: “Of these, 441 species (~70%) fulfilled the minimum requirement of five unique localities necessary for niche modeling using minimum volume ellipsoids, and 369 of those were present in the phylogenetic tree”.
I recommend stating the minimum occurrence requirement and resulting species counts in the Materials and Methods, and presenting results here directly for the final set of 369 analyzed species.

R: In our study, we estimated niche properties for 441 species. However, 369 of these were present in the phylogenetic tree and used exclusively to characterize the niche centroid of the most common recent ancestor (as indicated in the Methods section). To avoid confusion in the Results section, we modified the sentence. We retained the complete set of 441 species as our final sample size for all analyses, and we updated the manuscript accordingly to ensure consistency

Lines 261–262: “We obtained the potential distribution models for 441 treefrog species, while 711 species distribution polygons were obtained from IUCN”.
This sentence should be in the Methods, as all methodological decisions belong there. The Results section should present only outcomes.

R: We placed the information in the Methods Section, and we clarified the inclusion of this analysis as Reviewer 1 suggested.

There is inconsistency in sample numbers:
– abstract: “all American hylids analyzed”;
– line 128: 703 species compiled;
– line 253: 441 species reached the minimum threshold;
– line 369: species included in the phylogeny;
– line 261: 441 models + 711 IUCN polygons, exceeding the initial list of 703 species.
Also, each analysis should rely on the same number of independent species. Shouldn't amplitude, marginality, and position analyses all use the same 369 species?

R: We apologize for the confusion in these numbers. In our study, we estimated niche properties for 441 species. However, 369 of these were present in the phylogenetic tree and were used exclusively to characterize the niche centroid of the most recent common ancestor of the clade.

We changed “all American hylids analyzed” to *441 American treefrog species (70%)*.

We kept “703 species compiled” as they refer to the species list reported in the Americas from Amphibiaweb, which also serves as the taxonomic nomenclature used in our analyses.

Lines 316–317: “For the multidimensional approach, the ancestral reconstruction suggests that the 316 treefrog's ancestor centroid was at position pc1: 2.99, pc2: 0.85, pc3: 0.366”, and Lines 322–325: The multidimensional approach showed that the areas in The Americas that are near to the ancestral niche (i.e. 5% percentile) were in the Neotropical region and are associated to an annual mean temperature of 25°C and an annual mean precipitation of 2000 mm (S6)”. I suggest combining these sentences and standardizing the order of approaches.

R: We modified the text following your suggestion.

Line 349: “temperature and precipitation position”.
According to Table 2, precipitation position (PP) was not significant.

R: We apologize for the confusion in Table 2. We expressed significant values in scientific notation, for example, 5E-03 = 0.005. The coefficient of Precipitation position (PP) was significant in both models (p-values < 0.05). However, to avoid potential misunderstandings, we changed those values to <0.01.

Discussion
Lines 356–358: “Sites with high treefrog species richness in the Americas tend to be composed of species with narrower niche breadths and similar to that of their common ancestor”.According to Table 2, the richness–temperature-breadth relationship was not significant. Therefore, the statement that richness is higher in areas with narrower niches is not supported. Is that correct?
R: We apologize for potential misunderstandings in Table 2. According to our SAR models, all niche properties estimated under the multidimensional and unidimensional approaches were statistically significant (p-value < 0.05). To avoid potential misunderstandings, we changed those values to p<0.01.

Lines 356–359: “Sites with high treefrog species richness in the Americas tend to be composed of species with narrower niche breadths and similar to that of their common ancestor”. Specify that the result applies only to precipitation.
R: Thanks for pointing this out, we clarified the phrase as follows: *Sites with high treefrog species richness in the Americas tend to be composed of species with narrower niche breadths and similar to that of their common ancestor (i.e. low position), at least for the multivariate approach (i.e. ellipsoids) and precipitation.*

Lines 359–360: “Our results support the tropical niche conservatism hypothesis for the American treefrogs and provide clues as to the climatic conditions related to sites of origin”
Spatial autocorrelation did not indicate statistical significance for the richness × temperature breadth relationship.
R: All results from the SAR analyses that involved niche position, regardless of the approach (uni and multivariate), were statistically significant (p-value< 0.05; Table 2). However, the results for temperature position don’t agree with the expected negative relationship. We clarified this as follows: “*Results from the phylogenetic niche position analysis support the tropical niche conservatism hypothesis for the American treefrogs (except for temperature position), sites with high species richness contain species with niches like that of the ancestors and provide clues as to the climatic conditions related to sites of origin”.*

Line 368: “Climatic niche breadth was the only property that consistently exhibited a negative relationship with richness, whether it was based solely on temperature, precipitation, or a multivariate representation of climate”.
The statement includes negative but non-significant relationships, correct? In both the unidimensional (temperature) and multidimensional approaches. Niche breadth was supported only for precipitation in the unidimensional approach.

R: We apologize for potential misunderstandings concerning the results presented in Table 2. According to the SAR models, all niche properties estimated under the multidimensional and unidimensional approaches had a statistically significant effect on species richness (p-value < 0.05). To avoid potential misunderstandings, we changed the scientific notation to p<0.01

Lines 375–378: “SAR models indicate that niche breadth is negatively associated with species richness, nonetheless, the highest effect size on species richness was related to temperature breadth (Table 2)”. Again, this is a relationship not different from random expectations.
R: We apologize for potential misunderstandings concerning the results presented in Table 2. According to the SAR models, all niche properties estimated under the multidimensional and unidimensional approaches had a statistically significant effect on species richness (p-value < 0.05). To avoid potential misunderstandings, we changed the scientific notation to p<0.01.

Lines 380–383: “Interestingly, despite tropical latitudes exhibiting higher variation in precipitation than temperate regions [17], the sites with the highest variation in precipitation (e.g. Chocó and the Caribbean region) do not present high treefrog richness (Fig. 2c, 2f)”. Is there any possible ecological explanation for this result?

R: We consider an evolutionary explanation related to niche conservatism to be more likely than an ecological one. This is because the two regions (i.e. Pacific and Caribbean) exhibit contrasting precipitation regimes: the Caribbean region is characterized by low precipitation, whereas in the Pacific region, precipitation presents the highest regimes in the Americas. Given that the ancestral niche corresponds to intermediate precipitation levels, both regions occupy opposite extremes of the gradient (i.e. dry and wet) and are therefore far from the ancestral climatic conditions, which may contribute to their low species richness. This pattern could suggest an interaction between niche breadth and niche position. We briefly included this in discussion.

Other potential explanation to this result may be related to colonization time of other Anurans groups in these regions, which could limit the colonization of treefrogs. However, the only information that we found about the ancestral areas of treefrogs is inferred at a coarse scale (i.e. South America)

Line 397: “niches”.
Specify that this refers to temperature niches (not precipitation).

R: We changed as niche because we referred to temperature and precipitation as is specified in parenthesis.

Lines 420–422: “Interpretations of patterns or processes under the niche concept are more appropriate under a multidimensional perspective, which provides an overview of the species niche and not a single dimension”. It is unclear whether “multidimensional perspective” refers to combining unidimensional + multidimensional analyses or referring exclusively to multivariate analyses. If referring to the latter, note that the unidimensional approach provided more informative results. A multidimensional approach can also introduce noise into interpretation. For instance, the role of marginality remains unclear given its subjective nature.

R: When we mention the “multidimensional perspective” we refer to the multivariate analyses. We aim to highlight that the niche concept includes several ecological dimensions that affect species simultaneously, thus the interpretation of process under a niche perspective should include at least the joint effect of climate. However, we acknowledge in our discussion that both perspectives are informative, and that individual niche dimensions can provide valuable insight into the role of each dimension in explaining macroecological patterns.

We agree with the reviewer that a multidimensional approach may obscure certain patterns if a single niche dimension accounts for most of the variation underlying the estimation of multivariate properties
